# Metabolic Syndrome as a Cardiovascular Disease Risk Factor: Patients Evaluated in Primary Care

**DOI:** 10.1186/1471-2458-8-251

**Published:** 2008-07-22

**Authors:** Joan-Josep Cabré, Francisco Martín, Bernardo Costa, Josep L Piñol, Josep L Llor, Yolanda Ortega, Josep Basora, Marta Baldrich, Rosa Solà, Jordi Daniel, Josep Ma Hernández, Judit Saumell, Jordi Bladé, Ramon Sagarra, Teresa Basora, Dolors Montañés, Joan L Frigola, Angel Donado-Mazarrón, Maria Teresa García-Vidal, Isabel Sánchez-Oro, Josep M de Magriñà, Ana Urbaneja, Francisco Barrio, Jesús Vizcaíno, Josep M Sabaté, Irene Pascual, Vanesa Revuelta

**Affiliations:** 1 ABS Reus-1, Camí de Riudoms, 53–55, 43202 Reus, Spain; 2 ABS Reus-2, Camí de Riudoms 53–55, 43202 Reus, Spain; 3 ABS Deltebre, Avinguda Esportiva, 164, 43580 Deltebre, Spain; 4 ABS Reus-4, General Moragues 52, 43201 Reus, Spain; 5 ABS Reus-3, General Moragues 52, 43201 Reus, Spain; 6 Reus-Altebrat Primary Care Service, Spain; 7 Medicine Faculty, Rovira i Virgili University, Sant Llorenç 11, 43202 Reus, Spain

## Abstract

To estimate the prevalence of metabolic syndrome (MS) in a population receiving attention in primary care centers (PCC) we selected a random cohort of ostensibly normal subjects from the registers of 5 basic-health area (BHA) PCC. Diagnosis of MS was with the WHO, NCEP and IDF criteria. Variables recorded were: socio-demographic data, CVD risk factors including lipids, obesity, diabetes, blood pressure and smoking habit and a glucose tolerance test outcome. Of the 720 individuals selected (age 60.3 ± 11.5 years), 431 were female, 352 hypertensive, 142 diabetic, 233 pre-diabetic, 285 obese, 209 dyslipemic and 106 smokers. CVD risk according to the Framingham and REGICOR calculation was 13.8 ± 10% and 8.8 ± 9.8%, respectively. Using the WHO, NCEP and IDF criteria, MS was diagnosed in 166, 210 and 252 subjects, respectively and the relative risk of CVD complications in MS subjects was 2.56. Logistic regression analysis indicated that the MS components (WHO set), the MS components (IDF set) and the female gender had an increased odds ratio for CVD of 3.48 (95CI%: 2.26–5.37), 2.28 (95%CI: 1.84–4.90) and 2.26 (95%CI: 1.48–3.47), respectively. We conclude that MS and concomitant CVD risk is high in ostensibly normal population attending primary care clinics, and this would necessarily impinge on resource allocation in primary care.

## Background

The metabolic syndrome (MS) was first described in 1998 by Reaven as "syndrome X". Although previously alluded-to by several authors (Kylin, Marañón and others) and termed a multi-metabolic syndrome or insulin resistance, it represented a known risk factor in the development of cardiovascular disease events and was associated with accelerated atherosclerosis [1].

From the classical description by Reaven [2], a common nexus was established: insulin resistance/hyperinsulinemia. Surprisingly, this author did not include obesity in the definition. Later, other authors included the concept of alterations in fat, disorders of glucose, dyslipemias and hypertension. Recently, a growing interest has developed and MS has been receiving attention not only in hospital-based medicine but also in primary care (PC); albeit in the latter case the number of studies has been considerably lower. Since epidemiological studies suggest that > 25% of the general population will gradually develop insulin resistance [3] it would appear that this pathology will be diagnosed and treated increasingly within the ambit of PC [4-6] and, as such, has become included in the list of priorities of various public health-care authorities.

Apart from the difficulty in identifying its components, there is the added the lack of consensus diagnostic criteria. The initial criteria were those of the WHO in 1998 [7] and subsequently those of the NCEP-ATP III in 2001 which identified clinical types and simplified their management [8]. Other criteria of note are those of EGIR (European Group for the study of Insulin Resistance) based on European populations and were the first that defined prevalences in and around Spain. Subsequently, the American Association of Clinical Endocrinologists (AACE) developed their criteria in 2003 and, finally, the International Diabetes Federation (IDF) in 2005 issued its consensus document [9]. Irrespective of the criteria used, the certainty is that MS is very prevalent [10-12] and the initial studies in the USA recognize MS as a principal problem of health with developed countries, and with elevated social and economic consequences.

Reaven recognized some features frequently associated with MS (syndrome of polycystic ovaries, endothelial alteration and non-alcoholic hepatic steatosis)[13]. Of note is that the very existence of MS as a disease entity has been placed in doubt [14] such that the American Diabetes Association (ADA) and the European Association for the Study of Diabetes (EASD) recommended that patients are not to be labeled as MS. However, the IDF has published new criteria for MS adapted to different ethnic groups, among which is the European population [15].

The relationship between MS and cardiovascular disease has been well documented in the biomedical literature [16-21].

References in PC in Spain are available [22-28], and seems to highlight a high-risk population in our country, i.e. hypertension of 47.8% in some samples. In another investigation, the prevalence of MS was calculated as 50.6% overall in patients who had hypertension, dyslipemia, diabetes or a combination of these 3 elements.

As such, MS constitutes a multi-pathology frequently encountered in PC consulting rooms and its high prevalence would indicate the need for alertness in identifying the syndrome and to treatment its components in order to minimize the risk of subsequent cardiovascular disease.

## Objectives

1) To determine the prevalence of MS in the population receiving attention in PC.

2) To indentify factors or components of MS that independently influence the cardiovascular disease prognosis related to MS.

3) To analyse the impact of each component on the appearance of cardiovascular disease events.

## Methods

### a) Design

Prospective, multi-centered cohort study to observe the appearance of cardiovascular disease events. There were 2 cohorts: patients with the diagnosis of MS and those subjects without MS. Follow-up was for 2 years.

### b) Study population

Patients who were attending 5 Basic Health-care Areas (BHA) in Catalonia, Spain; 4 in the urban city of Reus and the 5^th ^located in the rural area of Deltebre. The catchment area of the PC centers has a total of approximately 72,000 individuals. The selection for the present study was patients of both genders aged above 45 years, and who had attended the clinical center at least once in the previous 3 years.

Using the Computerized System for Patient Care (CSPC) we selected a random list of individuals to reflect the distributions of the total of those subjects registered and assigned by the Governmental Health-service Authority to the different PC centers. Under the National Health Service provisions in Catalonia, all citizens are entitled to free-at-the-point-of-access medical attention which, under most circumstances, is the local PC center. The selected subjects were contacted and informed of the study and their written consent to participation was solicited. The objectives of the study were explained and, as an inducement, a thorough medical check-up and follow-up was offered. The tests included blood constituent analyses and a 75 g glucose tolerance test (except for known diabetics). All the blood samples were analyzed in the same hospital clinical laboratory which has international quality standard ceditation (ISO9002). Standard clinical history was taken and included information on pharmacological and non-pharmacological drugs used, advice on tobacco habit and on diet, and information with respect to MS diagnosis as well as any other possible illnesses. The exclusion criterion was that of having suffered any cardiovascular disease complication prior to recruitment to the present study. This item was evaluated from the clinical notes by 2 different members of the investigation team and, in case, of non-concordance, the opinion of a 3^rd ^member was sought.

### c) Study period

From 30^th ^June 2004 to 30^th ^June 2006.

### d) Information sources

We designed multi-parameter data collection forms on which the clinical history data were recorded at the initial interview as well as from the archived data recorded at each of the participating BHA centers. The items included; date of registration at the center, family and personal history, cardiovascular disease risk factors (hypertension, diabetes mellitus, dyslipemia, obesity), dietary and/or pharmacological treatments, blood constituent measurements, presence of vascular complications. The cardiovascular risk was calculated according to the 2 methods; the Framingham equation [29], and the REGICOR formula [30] (a calibration of Framingham algorhitm adapted for Spain) for each individual participant.

### e) Definition of variables

Age, gender, weight, height, body mass index (BMI), concentrations of plasma triglycerides, total cholesterol, HDL-cholesterol, LDL-cholesterol, uric acid, baseline insulinemia, baseline glycemia, glycemia at 2 h post-glucose load, fibrinogen, leukocyte count, glycated hemoglobin, waist-hip ratio, abdominal circumference, blood pressure, family history of cardiovascular disease and of diabetes, smoking habit, alcohol consumption, dietary and/or pharmacological treatment, presence of vascular complication as well as the date when these complications had been diagnosed.

### f) Definition of MS criteria and cardiovascular diseases

The criteria defining MS according to the NCEP-ATPIII require a combination of at least 3 of the 5 following criteria:

- Abdominal circumference ≥ 102 cm in males or ≥ 88 cm in females.

- HDL cholesterol < 1.03 mmol/mL (< 40 mg/dL) [males] or < 1.3 mmol/mL (< 50 mg/dL) [females].

- Triglycerides ≥ 1.7 mmol/mL (≥ 150 mg/dL).

- Blood pressure ≥ 130/85 mmHg or the patient receiving hypotensive treatment.

- Baseline glycemia > 6.1 mmol/mL (> 110 mg/dL).

Additionally, IDF criteria are the same precedent with the only differences of:

a) Abdominal circumference is, for European people, ≥ 94 cm in males or ≥ 80 cm in females.

b) Fasting glycemia > 5.55 mmol/l (> 100 mg/dL).

According to the WHO criteria, an individual is considered to have MS if:

- Glucose intolerance/diabetes/IFG and/or insulin resistance (determined with functional laboratory tests) plus:

- Two or more of the following criteria:

• Hypertension (or its treatment).

• Hypertriglyceridemia of > 1.7 mmol/mL (> 150 mg/dL) or LDL < 0.9 mmol/mL (< 35 mg/dL) [males] and < 1.0 mmol/mL (< 39 mg/dL) [females].

• BMI > 30 or WHI (waist-hip index) > 0.9 [males] or > 0.85 [females].

• Microalbuminuria (> 20 μg/min) or urinary albumin:creatinine ratio > 30 mg/g.

The patients who fulfilled the NCEP criteria for MS were entered into the study under as the MS cohort. The cohort defined as non-MS was composed of those patients that did not fulfill the NCEP criteria.

Cardiovascular disease complications were defined as ICD-9 codes corresponding to:

- Coronary heart disease: clinical history of ischemic heart disease (myocardial infarction, angina) and/or cardiac insufficiency, complementary assessments (ECG, stress test, scans...) (Codes 411 to 416).

- Cerebrovascular disease; clinical history suggestive of transitory ischemic attack/cerebrovascular accident and/or image tests that show such evidence (Codes 430 to 438).

- Peripheral vascular disease: intermittent claudication, absence of peripheral pulse or demonstrated by Doppler (ankle-brachial index < 0.9). (Codes 250.7, 443).

- Diabetic retinopathy: findings in the fundus of the eye compatible with the disease (Code 362).

- Nephropathy: microalbuminuria > 30 mg/24 h.

- Diabetic neuropathy: employing the Diabetic Neuropathy Symptom (DNS) scale that varies between 0–4 points, with neuropathy defined as a score of ≥ 1 point. Further corroboration with a pathology assessment using 10G-Semmes-Weinstein monofilament in one of more of the 6 plane areas evaluated.

- Also considered as a complication are those deaths related to cardiovascular disease e.g. myocardial infarction that causes death.

### g) Determination of sample size

With an α risk of 0.05 for a precision of ± 3% in a two-tailed test of difference in proportions of MS estimated as 17%, the requirement for a random population sample was 738 subjects; assuming the target population to be 36,600 individuals (36.6% of the total population defined). The loss rate was estimated at 20% (*Programa Granmo version 5.2, Institut Municipal d'Investigació Mèdica de Barcelona*) [31].

### h) Program of analysis

Epidemiological assessment: Possible associations between different variables or components of MS, with an increase in risk of having cardiovascular disease pathology.

Statistical analyses: the data obtained were analyzed using SPSS program (version 12.0) working with a confidence level of 95% and considering differences as being statistically significant at p < 0.05. Initially, a descriptive analysis was performed of the variables, and subsequent stepwise multivariate analyses were performed in order to determine the relative importance of the different risk factors as cause of the cardiovascular event. In the multivariate analyses, the dependent variable was the number of events and the independent variables were the dichotomized risk factors (hypertension, dyslipemia, diabetes, obesity). Also included was the dichotomized (presence/absence) variable of MS diagnosed on the WHO, NCEP and IDF criteria. The results are presented adjusted for age and gender, with respect to the variables that were significant in the model as well as the coefficients or the *b*-exponential that explain the percentage prediction of each variable considered in the analyses.

## Results

**Figure 1:**
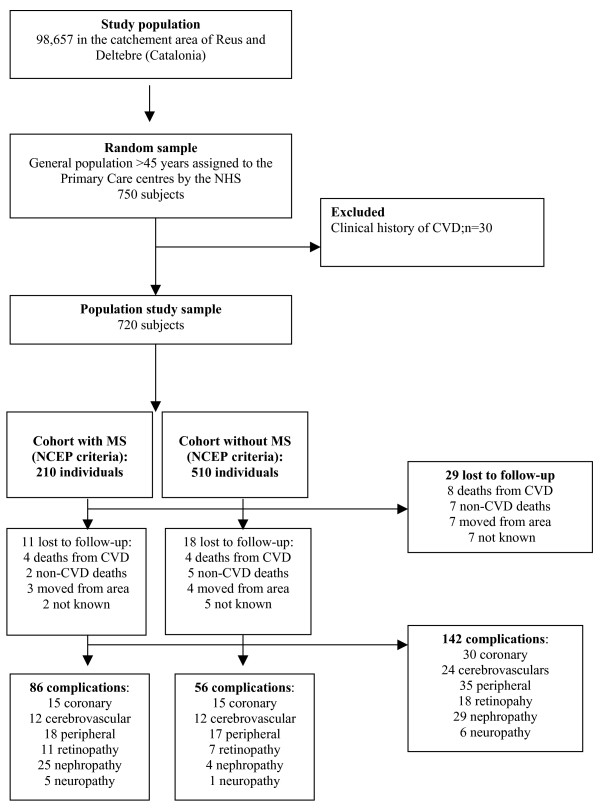
**Flow chart of the study:** Prospective, multi-centered cohort study to evaluate the cardiovascular disease event associated with metabolic syndrome in a randomly selected study sample.

Of the 750 subjects selected, 30 were excluded due to having had previous cardiovascular disease events. Of the 720 subjects followed-up, there were 29 (4%) losses: 8 cardiovascular disease deaths, 7 deaths from non-cardiovascular disease, 7 had moved out of the local area and 7 were lost to follow-up. Figure 1 describes flow of the study.

The overall mean age was 60.3 ± 11.5 years and 431 were female (59.9%). The risk factors were: hypertension in 352 (48.9%), diabetes type 2 in 142 (19.7%), pre-diabetes in 233 (32.4%), obesity in 285 (39.6%), dyslipemia in 209 (29%), and 106 (14.7%) were smokers. The mean BMI was 29.1 ± 5.2 kg/m^2^. The mean cardiovascular risk index (according to the Framingham score) was 13.8 ± 10% and, according to the REGICOR tables, was 8.8 ± 9.8%; both projected to 10 years.

With respect to MS, the study detected 166 subjects (23.1%; 95%CI: 20.0–26.3) who fulfilled the MS diagnostic criteria according to the WHO, and 210 (29.2%, 95%CI: 26.9–32.6) according to the NCEP-ATPIII criteria; 141 individuals fulfilled both definitions. 252 subjects (35.0%; 95%CI: 31.5–38.6) fulfilled IDF criteria; of them 210 fulfilled the NCEP criteria too. The *kappa *(κ) index of concordance between the first two sets of criteria defining MS was 0.66 (p < 0.001) and between the second two sets was 0.87 (p < 0.001).

Table 1 summarizes the information on the principal characteristics of the study sample segregated into two groups; without MS according to the NCEP criteria and those with MS according to the same criteria.

**Table 1:** Clinical and metabolic characteristics of the subjects as a function of the diagnosis of MS based on the NCEP-ATPIII criteria

	**TOTAL **(n = 720)	**Metabolic Syndrome (NCEP)**		**P**
		
		No (n = 510)	Yes (n = 210)	
Females	60.6(436)	51.4(262)	82.8(174)	**< 0.001**
Age;years	66.9(SD = 10.9)	63.2(SD = 11.2)	70.6(SD = 9.3)	**< 0.001**
**Obesity**	39.6(285)	22.3(114)	81.4(171)	**< 0.001**
BMI(kg/m^2^)	29.1(SD = 5.2)	27.7(SD = 4.6)	32.1(SD = 5.2)	**< 0.001**
FEMALES	27.9(SD = 6.0)	27.5(SD = 5.4)	33.5(SD = 4.9)	**< 0.001**
MALES	27.7(SD = 4.7)	27.1(SD = 3.9)	28.7(SD = 5.3)	**< 0.001**
Abdominal circumference(cm)	100.7(SD = 11.7)	95.8(SD = 13.0)	107.2(SD = 13.8)	< 0.03
**Blood pressure**	48.9(352)	36.1(184)	80.0(168)	**< 0.001**
BPS(mmHg)	132.0(SD = 17.6)	130.3(SD = 19.4)	142.0(SD = 18.7)	**< 0.001**
FEMALES	131.6(SD = 20.7)	127.4(SD = 19.3)	140.4(SD = 17.8)	**< 0.001**
MALES	133.4(SD = 18.1)	129.1(SD = 16.3)	145.6(SD = 18.7)	**< 0.001**
BPD(mmHg)	78.1(SD = 11.6)	78.4(SD = 11.7)	82.4(SD = 11.1)	**< 0.001**
FEMALES	78.5(SD = 11.7)	76.4(SD = 11.1)	81.6(SD = 10.5)	**< 0.001**
MALES	79.6(SD = 11.6)	79.0(SD = 11.0)	83.7(SD = 11.6)	**< 0.001**
**Diabetes mellitus**	19.7(142)	8.8(45)	60.5(127)	**< 0.001**
Baseline glycemia(mg/dL)	106.7(SD = 38.7)	102.1(SD = 31.7)	127.4(SD = 55.8)	**< 0.001**
FEMALES	111.4(SD = 42.3)	97.3 (SD = 27.5)	139.4(SD = 51.8)	**< 0.001**
MALES	114.8(SD = 46.0)	103.6(SD = 41.0)	133.5(SD = 48.1)	**< 0.001**
HbA_1c_;%	5.6(SD = 1.5)	5.4(SD = 1.3)	6.2(SD = 2.0)	**< 0.001**
FEMALES	6.04 (SD = 1.8)	5.32(SD = 1.2)	6.7(SD = 2.0)	**< 0.001**
MALES	6.11(SD = 1.9)	6.03(SD = 2.1)	6.2(SD = 1.6)	**< 0.001**
**Dyslipemia**	29.0(209)	16.1(82)	60.5(127)	**< 0.001**
Total cholesterol(mg/dL)	219.2(SD = 40.1)	217.3(SD = 42.5)	226.6(SD = 46.3)	**< 0.01**
FEMALES	219.6(SD = 41.5)	215.4(SD = 44.2)	239.7(SD = 52.6)	**< 0.001**
MALES	218.5(SD = 43.9)	217.9(SD = 43.9)	223.5(SD = 42.8)	**< 0.01**
LDL-cholesterol(mg/dL)	141.3(SD = 36.3)	140.9(SD = 35.9)	143.2(SD = 36.7)	**NS**
FEMALES	141.2(SD = 36.1)	139.8(SD = 36.3)	141.7(SD = 36.5)	**NS**
MALES	145.2(SD = 37.2)	143.3(SD = 35.8)	147.2(SD = 39.4)	**NS**
HDL-cholesterol(mg/dL)	54.4(SD = 19.0)	54.0(SD = 16.6)	50.8(SD = 17.4)	**< 0.001**
FEMALES	53.2(SD = 18.2)	58.7(SD = 18.4)	52.4(SD = 16.1)	**< 0.001**
MALES	50.9(SD = 22.3)	52.2(SD = 20.6)	50.1(SD = 20.8)	NS
Triglycerides(mg/dL)	151.4(SD = 109.6)	147.0(SD = 107.0)	165.4(SD = 118.3)	**< 0.001**
FEMALES	134.0(SD = 79.2)	104.4(SD = 59.2)	177.5(SD = 87.1)	**< 0.001**
MALES	174.0(SD = 135.7)	148.0 (SD = 123)	210.5(SD = 146.2)	**< 0.002**
Microalbuminuria(mg/d)	38.3(SD = 131.2)	18.2(SD = 64.1)	103.8(SD = 233)	**< 0.002**
FEMALES	17.8(SD = 42.3)	13.7(SD = 18.2)	24.5(SD = 64.1)	**< 0.001**
MALES	65.5(SD = 191.3)	42.5(SD = 112.4)	101.7(SD = 269)	**< 0.001**
**CVD risk**(% at 10 years)	13.8(SD = 10.0)	13.4(SD = 8.3)	23.8(SD = 10.0)	**< 0.001**
FEMALES	8.7(SD = 7.9)	6.5(SD = 5.3)	17.3(SD = 10.0)	**< 0.001**
MALES	19.7(SD = 11.1)	14.4(SD = 9.8)	26.4(SD = 11.6)	**< 0,001**
**REGICOR**(% at 10 years)	8.8(SD = 9.8)	7.2(SD = 8.9)	10.7(SD = 9.5)	< 0.001
FEMALES	3.7(SD = 5.3)	2.9(SD= 4.7)	5.6(SD = 7.1)	**< 0.001**
MALES	10.1(SD = 8.8)	8.2(SD = 9.0)	13.5(SD = 9.4)	**< 0.001**
**CVD complications**	19.7(142) 113 individuals	11.0(56) 55 individuals	40.9(86) 58 individuals	< 0.001
Cerebrovascular	3.3(24)	2.3(12)	5.7(12)	< 0.001
Coronary	4.2(30)	2.9(15)	7.1(15)	< 0.001
Peripheral vascular	4.9(35)	3.3(17)	8.6(18)	< 0.001
Retinopathy	2.5(18)	1.4(7)	5.2(11)	< 0.02
Nephropathy	4.0(29)	0.8(4)	11.9(25)	< 0.001
Neuropathy	0.8(6)	0.2(1)	2.4(5)	< 0.001

The data are expressed in percentages (absolute values) and mean (SD:standard deviation). BPS = systolic blood pressure; BPD = diastolic blood pressure; BMI = body mass index; CVD = cardiovascular disease risk according to the Framingham predictive equation; REGICOR: cardiovascular disease risk tables based on the Framingham equations and calibrated for Spain; NS = not significant

Cardiovascular disease complications appeared in 113 (15.7%) subjects during the follow-up of 2 years. Overall, there were 142 different complications: 35 (4.9%) were peripheral vascular disease, 30 (4.2) coronary artery disease, 29 (45) nephrotic syndrome, 24 (3.3%) cerebrovascular disease, 18 (2.5%) classified as having retinopathy and 6 (0.8%) developed neuropathy. Segregated by cohort, the group with MS suffered 15 coronary complications, 12 cerebrovascular, 18 peripheral artery disease, 11 retinopathies, 25 nephropathies and 5 neuropathies. Conversely, in the group without MS, the complications were 15 coronary disease, 12 cerebrovascular, 17 peripheral vascular disease, 7 retinopathies, 4 nephropathies and 1 neuropathy.

Forward stepwise multiple logistic regression analyses of the factors that influenced the appearance of the cardiovascular disease events are summarized in Table 2 and from which it is of note that each MS classification carries a different weight for different complications. As observed in the multivariate analyses (Table 2) the WHO criteria are better predictive of cardiovascular disease event than those of the IDF. This is evident when the complications are considered globally (OR = 3.48) as well as individually (i.e. cases of coronary artery disease, vascular disease and nephropathy). Conversely, the NCEP criteria predict the cerebrovascular, arteriopathy, nephropathy and neuropathy complication. IDF criteria predict the globally complications, as well as coronary, retinopathy and nephropathy.

**Table 2:** Logistic regression of component factors in cardiovascular disease risk, adjusted for age and gender.

CVD complication	B exponential = Odds Ratio(95%CI)*
All complications	Females;OR = 2.26(1.48–3.47)
	Metabolic syndrome (WHO criteria);OR = 3.48(2.26–5.37)
	Metabolic syndrome (IDF criteria);OR = 2.28(1.84–4.90)

Coronary	Metabolic syndrome (WHO criteria);OR = 3.10(1.48–6.49)
	Metabolic syndrome (IDF criteria);OR = 1.96(1.15–4.89)

Cerebrovascular	Females;OR = 2.46(1.06–5.37)
	Metabolic syndrome (NCEP criteria);OR = 2.59(1.26–5.27)

Peripheral vascular	Females; OR = 3.61(1.72–7.55)
	Metabolic syndrome (WHO criteria);OR = 2.26(1.11–4.61)
	Metabolic syndrome (NCEP criteria);OR = 2.74(1.82–5.33)

Retinopathy	Metabolic syndrome (NCEP criteria);OR = 2.80(1.07–7.34)
	Metabolic syndrome (IDF criteria);OR = 3.63(2.27–7.94)

Nephropathy	Females; OR = 2.97(1.32–6.66)
	Metabolic syndrome (WHO criteria); OR = 11.78(4.91–28.3)
	Metabolic syndrome (IDF criteria);OR = 3.51(2.07–12.2)

Neuropathy	Metabolic syndrome (NCEP criteria);OR = 12.41(1.44–106.9)

* For each component are indicated those components included in the regression equation. The rest of the components did not reach statistical significance and, as such, have not been included in the table

The χ^2 ^analysis of the number of components of MS (NCEP criteria) indicated significant differences (p < 0.001) between the subjects with 0, 1 or 2 components (absence of MS) and those that had 3, 4 or 5 (presence of MS). The prevalence of events was 10.78% in subjects free of MS and 27.61% in subjects suffering from MS (OR = 2.56).

The differences were, as well, significant with the χ^2 ^test (p < 0.001) between individuals who did, and did not, fulfill the criteria using the WHO criteria. The prevalence of CVD events was 10.8% in the group without MS and 31.9% in the group with MS (OR = 2.95).

The rate of cardiovascular disease events during the first 2 years of follow-up was 98.6 events/1,000 patient-years.

**Figure 2:**
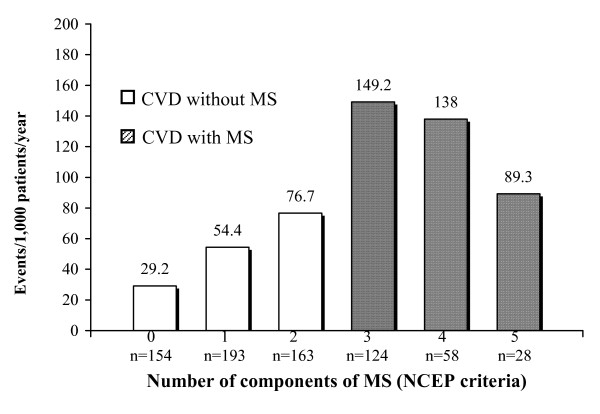
Cardiovascular disease (CVD) events segregated with respect to number of components of metabolic syndrome (MS); NCEP criteria.

In Figure 2 describes the increasing number of events per 1,000 inhabitants/year segregated with respect to the number of MS components (NCEP criteria).

**Figure 3:**
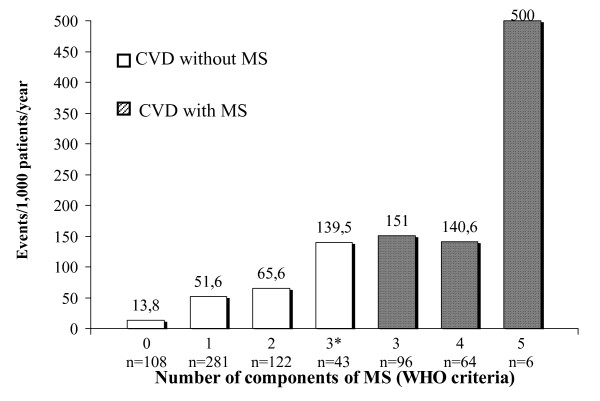
Cardiovascular disease (CVD) events segregated with respect to number of components of metabolic syndrome (MS): WHO criteria; * = alterations in glucose metabolism not included in the components.

Figure 3 depicts the progressively increasing numbers of events in relation to the number of MS components (WHO criteria).

**Figure 4:**
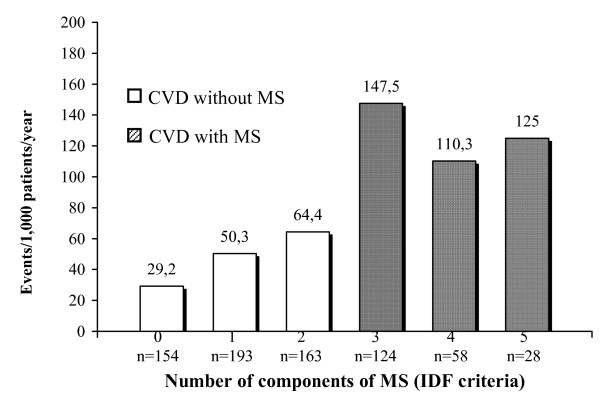
Cardiovascular disease (CVD) events segregated with respect to number of components of metabolic syndrome (MS); IDF criteria.

Figure 4 describes the number of events in relation to the number of MS components (IDF criteria).

In a sub-analysis of the patients without diabetes (n = 578) we observed that 93 had NCEP criteria for MS and, by the end of the follow-up period, these cases developed 16 (17.2%) complications. Conversely, in the subgroup of 485 subjects without MS or diabetes, there were a total of 43 (8.9%) cardiovascular disease complications.

Considering the diabetic patients alone (n = 142), 97 fulfilled the NCEP criteria for MS and, at 2 years of follow-up, there were 62 (63.9%) CVD complications compared to 21 in the 45 (46.6%) diabetic individuals without MS.

## Discussion

In primary care, the prevalence observed of MS is high and close to the values described in the worldwide literature, representing a problem of health at the developed countries.

After adjusting for age and gender, the analyses performed suggest that MS by WHO and IDF criteria are better predictive of cardiovascular disease events (as independent factor) than those of the NCEP (as well the complications are considered globally as well as individually). Individually, WHO criteria predicts better coronary artery disease, vascular disease and nephropathy; NCEP criteria the stroke, arteriopathy, nephropathy and neuropathy; and finally the IDF set predict globally complications, as well as coronary, retinopathy and nephropathy.

Of note is that there is a high prevalence of type 2 diabetes and of pre-diabetes in our sample. This is because a glucose tolerance test was performed in all study subjects (except those with known diabetes); the objective being to discount previously-unidentified alterations of carbohydrate metabolism. This criterion provides added value to the study since the prevalence identified is closer to the reality. The total of diabetes and pre-diabetes was > 50% of the study sample and which is of considerable note for the future disease development.

The principal difficulty in the diagnosis of MS is the criteria used. We followed the criteria of the NCEP-ATPIII which are more clinically-based than those of the previous WHO criteria of 1998. However, we performed the calculation based on the latest methodology as well. We did not apply other diagnostic methods such as those of the EGIR nor the AACE because the recommended tests were tests are required laboratory facilities that are beyond our reach (and that of most PC centers) such as analyses demonstrating insulin resistance.

The concordance between the WHO and NCEP criteria (*kappa *index of 0.66) and between the NCEP and IDF criteria (*kappa *index of 0.87) provides weight to our procedures and indicates that, in usual-care clinical practice it is not important which set of criteria are used to assess MS but more importantly to be aware of MS and to detect its components.

With respect rest to other methodological aspects, primary health care provision is distributed according to the risk factor status of the individual. The first clinical consultation would detect some aspects such as obesity and hypertension and which require prompt action (confirmation of diagnosis, dietary advice, life-style modifications) all of which are standard in our type of clinical practice. In the present study the added benefit for our general population (and of other similar populations) is that of screening. We were able to detect pathologies which we were not aware of (e.g. identification of subject with hyperglycemia or hypertension) and to detect previously-unknown risk factors for CVD. Further, we were able to provide advice on life-style modifications, such as anti-smoking, diets, exercise and body-weight control for what is an ostensibly healthy population.

Further, the identification of patients with MS creates an overall "attitude" for the individual in question such that the idea is developed that the preoccupation should not be towards only certain risk factors but, more importantly, all components that have an impact of future CVD.

However, we need to reiterate that we did not conduct an intervention study. Indeed, the measures we employed are standard in PC practice.

The association of MS with cardiovascular disease observed in the present study (OR = 2.56) is comparable with an epidemiological study conducted in the USA using a questionnaire on health (NHANES III) and in which an odds ratio of 2 was calculated not only for myocardial infarction but also for cerebrovascular disease (stroke) in both genders, and independently of age and ethnicity [33].

The prevalence in the USA now is slighty higher comparing data with newest NHANES from 1999–2000, increasing up to 27.0% [34]. Considering all the criteria set available nowadays, all of them are capable to predict cardiovascular disease, in differents degrees [35]. For example, stroke was predicted, in a 14-year follow-up in Finland, better by WHO and NCEP set than other criteria used [36].

The global prevalences in another European studies are closer: in Italy, finding a 16.2% but in youngest population [37]; in Greece in a nationwide survey, reaching a 23.6% [38] and in Great Britain finding a 29.8% (in women, with highest mean age) [39]. In Germany was lowest by NCEP definition (19.8%) but highest using the IDF definition (32.7%) [40].

So, the prevalence is increasing with each newly published definition of the MS and clearly associated with age and gender, as these studies shows.

Figure 2 shows that the CVD complications had been more numerous in the sub-group of subjects who had 3 components of MS, compared to those who had 4 or 5. This can be explained by the small numbers of complications in the sub-groups. Figure 3 shows that the patients who fulfilled 5 criteria on the WHO scale (the maximum) had a rate of events that was almost triple that of other sub-groups. The explanation in this case is that, apart from being relatively few in absolute numbers, the WHO criteria requires microalbuminuria as part of the MS diagnosis, and which is a known independent risk factor for cardiovascular disease. Figure 4 shows CVD complications similar to the Figure 2.

We believe, as well, that the better predictor of cardiovascular disease achieved by the WHO criteria over those of the NCEP (Table 2) needs to take microalbuminuria into account. The WHO criteria achieve a better prediction in overall CVD events (coronary, nephropathy). Conversely, the NCEP criteria is associated with a better prediction of stroke and arteriopathy. And finally, IDF criteria is a good predictor of retinopathy, nephropathy, coronary disease and all events globally. With respect to concordance between criteria used (*kappa *index of 0.66 between WHO and NCEP; and 0.87 between NCEP and IDF) we propose that the clinical effort should be applied to identifying the patients with high risk, irrespective of the choice of criteria used.

Undoubtedly, age and diabetes *per se *adds an important cardiovascular disease risk in all cases. Indeed, we need to highlight that in this sub-group, the MS modulates the risk such that those diabetic subjects with MS suffer more complications (63.9% vs. 46.6%) than those diabetic subjects without MS. Conversely, in the sub-analyses which excluded the patients with diabetes, and not taking the presence of diabetes into the definition of MS, the prediction of cardiovascular disease events was greater globally when MS was present (17.2% vs. 8.9%).

## Conclusion

In conclusion, we believe that MS diagnosis is simple and easy clinical tool to assess potential cardiovascular risk and, as such, can identify those patients who can benefit most from the prioritization of health-care resources, and we conclude that MS and concomitant CVD risk is high in ostensibly normal population attending primary care clinics.

## Competing interests

The authors declare that they have no competing interests.

## Authors' contributions

JC, FM, BC, JP, JB and RS participated in the design of the study. BC conceived of the study, and participated in its coordination. JP performed the statistical analysis. JC, FM, JL, YO, TB, DM, IS-O, AD-M, JF, MG-V, MB, RS, AU, JM, JB and JS included the patients. FB, JV, JS, JH, IP, VR, JB and JS revised the clinical outcomes. All authors read and approved the final manuscript.

## Pre-publication history

The pre-publication history for this paper can be accessed here:



## References

[B1] Isomaa B, Almgren P, Tuomi T, Forsén B, Lahti K, Nissén M (2001). Cardiovascular morbidity and mortality associated with the metabolic syndrome. Diabetes Care.

[B2] Reaven GM (1988). Role of insulin resistance in human disease. Diabetes.

[B3] Rupp H (1992). Insulin resistance, hyperinsulinaemia, and cardiovascular disease. The need for novel dietary prevention strategies. Basic Res Cardiol.

[B4] McAuley KA, Williams SM, Mann JI, Walker RJ, Lewis-Barned NJ, Temple LA (2001). Diagnosing insulin resistance in the general population. Diabetes Care.

[B5] Gerstein HC, Anand S, Yi QL, Vuksan V, Lonn E, Teo K (2003). The relationship between dysglycemia and atherosclerosis in South Asian, Chinese, and European individuals in Canada: a randomly sampled cross-sectional study. Diabetes Care.

[B6] Costa B, Cabré JJ, Martín F (2003). Metabolic syndrome, insulin resistance and diabetes. What is hidden beneath the tip of the iceberg?. Aten Primaria.

[B7] Alberti KGMM, Zimmet PZ, for the WHO Consultation (1998). Definition, diagnosis and classification of diabetes mellitus and its complications. Part 1: Diagnosis and classification of diabetes mellitus, provisional report of a WHO consultation. Diabet Med.

[B8] Executive Summary of the Third report of the National Cholesterol Education Program (NCEP) (2001). Expert Panel on detection, evaluation, and treatment of high blood cholesterol in adults (Adult Treatment Panel III). JAMA.

[B9] Grundy SM, Cleeman JL, Daniels SR, Donato KA, Eckel RH, Franklin BA (2005). American Heart Association. National Heart, Lung, and Blood Institute. Diagnosis and management of the metabolic syndrome: an American Heart Association/National Heart, Lung, and Blood Institute Scientific Statement. Circulation.

[B10] Serrano M, Ascaso JF, Blázquez E, Cabezas J, Cármena R, Escobar F (2002). Grupo de Trabajo Resistencia a la insulina de la Sociedad Española de Diabetes. Insulin resistance and its involvement in multiple risk factors associated with type 2 diabetes mellitus. Med Clin (Barc).

[B11] Balkau B, Charles MA, Drivsholm T, Borch-Johnsen K, Wareham N, Yudkin JS, for The European Group for the Study of Insulin Resistance (EGIR) (2002). Frequency of the WHO metabolic syndrome in European cohorts, and an alternative definition of an insulin resistance syndrome. Diabetes Metab.

[B12] Ford ES, Giles WH, Dietz WH (2002). Prevalence of the metabolic syndrome among US adults. JAMA.

[B13] Reaven GM (2002). Metabolic syndrome: pathophysiology and implications for management of cardiovascular disease. Circulation.

[B14] Kahn R, Buse J, Ferrannini E, Stern M (2005). American Diabetes Association; European Association for the Study of Diabetes. The metabolic syndrome: time for a critical appraisal: joint statement from the American Diabetes Association and the European Association for the Study of Diabetes. Diabetes Care.

[B15] Albert KGMM, Zimmet P, Shaw J (2005). The metabolic syndrome: a new worldwide definition. The Lancet.

[B16] Malik S, Wong ND, Franklin SS, Kamath TV, L'Italien GJ, Pio JR (2004). Impact of the metabolic syndrome on mortality from coronary heart disease, cardiovascular disease, and all causes in United States adults. Circulation.

[B17] McNeill AM, Rosamond WD, Girman CJ, Golden SH, Schmidt MI, East HE (2005). The metabolic syndrome and 11-year risk of incident cardiovascular disease in the Atherosclerosis Risk in Communities study. Diabetes Care.

[B18] Sattar N, Gaw A, Scherbakova O, Ford I, Lowe GD, O'Reilly DS (2003). Metabolic syndrome with and without C-reactive protein as a predictor of coronary heart disease and diabetes in the West of Scotland Coronary Prevention study. Circulation.

[B19] Alexander CM, Landsman PB, Teutsch SM, Haffner SM (2003). Third National Health and Nutrition Examination Survey (NHANES III); National Cholesterol Education Program (NCEP). NCEP-defined metabolic syndrome, diabetes, and prevalence of coronary heart disease among NHANES III participants age 50 years and older. Diabetes.

[B20] Lakka HM, Laaksonen DE, Lakka TA, Niskanen LK, Kumpusalo E, Tuomilehto J (2002). The metabolic syndrome and total and cardiovascular disease mortality in middle-aged men. JAMA.

[B21] Wannamethee SG, Shaper AG, Lennon L, Morris RW (2005). Metabolic syndrome vs Framingham Risk Score for prediction of coronary heart disease, stroke, and type 2 diabetes mellitus. Arch Intern Med.

[B22] Rigo F, Frontera J, Llobera J, Rodríguez T, Borrás I, Fuentespina E (2005). Prevalence of cardiovascular risk factors in the Balearic Islands (CORSAIB Study). Rev Esp Cardiol.

[B23] Anonymous (2000). Prevention of coronary heart disease in clinical practice-Summary of recommendations. Rev Esp Cardiol.

[B24] Alvarez LA, Suárez C, Mantilla T, Franch J, Ruilope LM, Banegas JR (2006). PREVENCAT Study: control of cardiovascular risk in primary care. Med Clin (Barc).

[B25] Tomás L, Varas C, Pérez I, Puig T, Balaguer I (2001). Risk factors and coronary morbimortality in a Mediterranean industrial cohort over 28 years of follow-up. The Manresa Study. Rev Esp Cardiol.

[B26] González JR, Alegría E, Lozano JV, Llisterri JL, García JM, González I (2001). Impact of hypertension in cardiac diseases in Spain. The CARDIOTENS Study 1999. Rev Esp Cardiol.

[B27] Pineda M, Custardoy J, Andreu MT, Ortín JM, Cano JG (2002). Grupo de Investigación Clínica del Sureste (GICS). Prevalence study of cardiovascular risk factors in a health area. Aten Primaria.

[B28] Alegría E, Cordero A, Laclaustra M, Grima A, León M, Casasnovas JA (2005). Prevalence of metabolic syndrome in the Spanish working population. MESYAS registry. Rev Esp Cardiol.

[B29] Anderson KM, Wilson PWF, Odell PM, Kanell WB, Framingham (1991). An update coronary risk profile. A statement for health professionals. Circulation.

[B30] Marrugat J, Solanas P, D'Agostino R, Sulllivan L, Ordovás J, Cordón F, Kannel WB (2003). Coronary risk estimation in Spain using a calibrated Framingham function. Rev Esp Cardiol.

[B31] Marrugat J, Vila J, Pevesi P, Sanz F (1998). Estimation of the sample size in clinical and epidemiological investigations. Med Clin (Barc).

[B32] IDF The IDF consensus worldwide definition of the metabolic syndrome. Berlin.

[B33] Ninomiya JK, L'Italien G, Criqui MH, Whyte JL, Gamst A, Chen RS (2004). Association of the Metabolic Syndrome With History of Myocardial Infarction and Stroke in the Third National Health and Nutrition Examination Survey. Circulation.

[B34] Ford ES, Giles WH, Mokdad AH (2004). Increasing prevalence of the metabolic syndrome among U.S. adults. Diabetes Care.

[B35] Wang J, Li HB, Kinnunen L, Hu G, Jarvinen TM, Miettinen ME, Yuan S, Tuomilehto J (2007). How well does the metabolic syndrome defined by five definitions predict incident diabetes and incident coronary heart disease in a Chinese population?. Atherosclerosis.

[B36] Wang J, Ruotsalainen S, Moilanen L, Lepistö P, Laakso M, Kuusisto J (2008). The metabolic syndrome predicts incident stroke. A 14-year follow-up study in elderly people in Finland. Stroke.

[B37] Moebus S, Hanisch JU, Aidelsburger P, Bramlage P, Wasem J, Jöckel KH (2007). Impact of 4 different definitions used for the assessment of the prevalence of the Metabolic Syndrome in primary healthcare: the German Metabolic and Cardiovascular Risk Project (GEMCAS). Cardiovascular Diabetology.

[B38] Athyros VG, Bouloukos VI, Pehlivanidis AN, Papageorgiou AA, Dionysopoulou SG, Symeonoidis AN (2005). The prevalence of the metabolic syndrome in Greece: The MetS-Greece Multicentre Study. Diabetes, Obesity and Metabolism.

[B39] Mancia G, Bombelli M, Corrao G, Facchetti R, Madotto F, Giannattasio C (2007). Metabolic syndrome in the Pressioni Arteriose Monitorate E Loro Associazioni (PAMELA) study: daily life blood pressure, cardiac damage, and prognosis. Hypertension.

[B40] Lawlor DA, Smith GD, Ebraihm S (2006). Does the new International Diabetes Federation definition of the metabolic syndrome predict CHD any more strongly than older definitions? Findings from the British Women's Heart and Health Study. Diabetologia.

